# Editorial Notes

**Published:** 1882-05

**Authors:** 


### EDITORIAL NOTES.

Sugar-Coated Pills—The prejudice against this form of coating is being overcome by the superior preparation of Warner & Co.'s Pills. Quinine Pills a year old show a soft and easily soluble interior on being cut open. Their pills are in every case reliable, so far as our experience with them goes.

Medical Colleges—Louisville, with a population not greater than Buffalo, has *five*. How superior these western towns are in medical knowledge. Buffalo has to call in the professional talent of New York, Hartford, Rochester and Norwich to supply professors for one, although we have as many learned and successful practitioners here as in any eastern city of equal size.

Glonoine—The *Journal* called attention to the potency of this new remedy nearly two years ago, and now its value is being very generally recognized. It ranks seconds only to digitalis in the treatment of cardiac disease. It is especially indicated in angina pectoris and weak dilated and fatty heart. In the latter it gives relief by reducing arterial tension and thus lessening the amount of work the heart has to do.

The *American Monthly Microscopical Journal* is published at 53 Maiden Lane, New York. Subscription $1.00 a year. Every physician who uses a microscope should be a subscriber. The publishers say that a comparatively small number of medical men receive the *Journal*. We believe it is because its value, or even its name, is unknown to very many practitioners. We can only say that any one who subscribes for it, will get more than full value for their money. It is one of our most valued exchanges.

Euthanasia—A good deal has been written in favor of the propriety and wisdom, giving those poor victims afflicted with incurable and painful diseases a comfortable send-off to Paradise. A missionary among the poor in London put the theory into practice, and when he found his parishioners suffering with cancer and other incurable diseases, and without the means to procure necessaries for their comfort, at the sick persons request, would administer a dose of morphia sufficient to put them into a sleep which knew no waking. Unfortunately the jury before which he was tried were not believers in the theory, and he was convicted of murder in the second degree and transported for life for thus relieving incurable suffering.

